# Impacts of Pretreatment Techniques on the Quality of Tuber Flours

**DOI:** 10.1155/2022/9323694

**Published:** 2022-06-27

**Authors:** Abebe Desalegn Melese, Ebisa Olika Keyata

**Affiliations:** ^1^Department of Chemical Engineering, Faculty of Technology, Shambu Campus, Wollega University, Shambu Town, Ethiopia; ^2^Department of Food and Nutritional Sciences, Faculty of Agriculture, Shambu Campus, Wollega University, Shambu Town, Ethiopia

## Abstract

Besides dietary sources of energy, roots and tuber crops can also serve as functional foods and nutraceutical ingredients to be explored in disease risk reduction and wellness. However, they are easily spoiled because of their high moisture contents and enzymatic reactions. Therefore, this review aimed at gathering information on the effects of various pretreatment methods on the quality of tuber crops before converting them into flour. Studies reported by different scholars showed that there were significant differences in physicochemical and functional properties between untreated tuber and the treated tuber samples. The review also highlighted that the chemical treatment methods, particularly sulfite treatment, could increase the lightness value of the flour. In addition, blanching could induce a decrease in protein, ash, and fat. Despite this, blanching pretreatment techniques increased moisture and carbohydrate content. Chemical treatment increases the ash content, which might be responsible for chemical diffusion into the sample. The reviewed results showed that the application of different pretreatments on tuber crops significantly improves many quality characteristics such as color, fiber content, carbohydrate, and the functional properties used for value addition during food product development in the industry. Therefore, application of pretreatment methods particularly chemical treatments could enhance nutritional value, and functional and physical properties of tuber flours.

## 1. Introduction

Root and tuber crops are grown in hot and humid regions worldwide [1]. In developing countries, tuber crop production serves as source of both food and income [2]. Among tuber crops, potato (*Solanum tuberosum Lam.)*, Oromo dinnich (*Plectranthus edulis*), sweet potato (*Ipomoea batatas* L., *Lam.*), anchote (*Coccinia abyssinica Lam., Cogn*.), cassava (*Manihot esculenta*), and yam (*Dioscorea bulbifera*, *Dioscorea rotundata*) are widely cultivated in Ethiopia.

Potato is a starch crop, which is consumed by more than one billion people in the world and the third most important food crop in the world after maize, wheat, and rice [3]. Cassava is the main source of dietary food energy for the majority of people living in the sub-humid tropics of West and Central Africa [4]. Aerial yam is one of the most important consumed staple steam starch tuber crops and is used as income generation for millions of people in many tropical and subtropical countries. Rather, in the case of nutrition content, it has an appreciable source of protein, vitamins, and minerals [5, 6]. Anchote is a nutritious starchy tuberous crop indigenous to Ethiopia particularly, the southwest part of the country. It contains substantial nutrients such as carbohydrates, protein, fiber, and different minerals mainly calcium [7]. Oromo potato is an indigenous stem tuber crop that is a good source of carbohydrates and proteins and also used as a source of income generation and food security in south and south western parts of the country [8].

The tuber crops indicated above were produced in huge quantities due to their adaptability to various climatic conditions, grown in different types of soil, and disease resistance. However, the perishable characteristics of the tuber crops limit the production interest of the growers. Additionally, the high moisture contents and ease of bruising reduce the shelf life of tuber crops during storage and production. Capotorto et al. [9] also reported that inappropriate harvesting and poor post-harvest handling are factors that cause physical damage, make tuber crops susceptible to fungal attack, and also increase the rate of dehydration and browning.

To enhance the shelf life of tuber crops, it is essential to inactivate enzymes and reduce the water activity through various pretreatment techniques including chemical, thermal, and physical methods [10]. Blanching is one of the most important pretreatment methods, which is used to inactivate polyphenol oxidase (PPO) enzymes through mild heat. It can modify texture, maintain color, flavor, and nutritional value, and eliminate trapped air. Commonly, the blanching industries adopt hot water and steam blanching heating media depending on the nature of the crops [11, 12]. Likewise, soaking is another pretreatment method used to retard the enzymatic browning reactions. There are various methods involving soaking solutions that are usually performed to avoid browning such as sodium metabisulfite, table salt (NaCl), and citric acid or ascorbic acid [9, 13].

After pretreatment, it is imperative to process the tuber crops into flour for further utilization as a value addition during new food product development. The food products made from tuber crop flour are major parts of the staple diet for most developing countries; hence, the ability to form composite flour and the availability of the flour are major economic issues [14]. Incorporation of flours from tuber crops into various products together with other cereal flours and improvements in the quality characteristics of such composite flours have been reported [15]. Different scholars have reported that the potentials of tuber flours during various food product developments such as bread, cookies, noodles, pretzels, cakes, porridges, and injera (an Ethiopian bread made from teff flour) to enrich nutrients, diversify food products and replace wheat flour to reduce the hard currency in a low-income country [16–19]. Encouragement and understanding the existing situation regarding the impacts of pretreatment techniques on the quality of tuber flours on the physicochemical properties of tuber crops are essential for utilization of tuber flours as value addition in the food industry. However, there are limited comprehensive reviews in Ethiopia regarding the impact of different pretreatments on tuber crops to inhibit oxidation and enzymatic reactions. Therefore, this review was aimed to assess scholars' suggestions concerning the main impacts of pretreatment techniques on the quality of tuber flours.

## 2. Impacts of Pretreatments on Quality Characteristics of Tuber Flours

In this review, factors that determine the quality of tuber flours after pretreatments such as physical properties, proximate composition, and functional properties are highlighted in the following paragraphs:

### 2.1. Pretreatment Effect on the Physical Properties of Tuber Crops

Data collected from different scholars regarding the physicochemical properties are summarized in Table 1.

#### 2.1.1. Particle Size

Particle size is an important property that may influence the physicochemical properties of tuber flours such as swelling, water binding capacity, and pasting properties [25]. Harijono et al. [26] reported that blanching treatment by using hot water reduces the starch granule size of the purple water yam flour from 19–46 *µ*m (nonblanched) to 17–38 *µ*m (blanched). The reduction of starch granules is advantageous because it increases its digestibility [27].

However, yam blanched using hot water relatively produced greater particle sizes (50% of the blanched flour particles were less than 100 *μ*m) compared to the control and sodium metabisulfite-treated flours (70% of the control and sodium metabisulfite-treated samples were below 100 *μ*m) [20]. This could be due to the pregelatinization of the starch granules generated by blanching, which stiffened and became more difficult to break into finer particles after drying [28]. Generally, flours of smaller particle size (below 130 *µ*m), mainly those treated with sodium metabisulfite, were suitable in applications of bakery such as baking bread, biscuits, and other pastry products [29].

#### 2.1.2. pH

Pretreatment and drying methods had a significant effect on the pH of sweet potato flour samples, which ranged from 4.5 to 7.0 [16]. Among the types of pretreatments applied by these authors, citric acid treatment increased the acidity of flour samples by decreasing pH values, acting as an acidifying agent. On the other hand, a salt treatment could increase the pH value. Ngoma et al. [21] reported that the untreated sweet potato flour samples recorded the highest pH value than the other treatment methods. The citric acid and sodium metabisulfite pretreatments could reduce the pH values of the flour; as their concentration increases, the acidity of the flour increases. The low pH values might be due to high amylase activity which was responsible for the increase in acidity.

Commonly, low pH value imparts a sour taste to the flour, which makes it less preferable for consumption [30]. However, acidic flours have a lower risk of deterioration by microbial spoilage, enzymes, or no enzymatic reactions [31]. Very acidic ﬂours (pH < 4) may result in considerable extension of fermentation and hence extended starch breakdown. Those acidic ﬂours are not suitable for processing into bakery and pastry products [32].

#### 2.1.3. Color Value

Color is an essential physical quality factor directly related to the acceptability of food products and helps the consumers to make purchase decisions of bakery products. The Commission Internationale d'Eclairage (CIE) created the *L*^*∗*^*a*^*∗*^*b*^*∗*^color space in 1976 as an international standard for color measurement. The *L*^*∗*^*a*^*∗*^*b*^*∗*^color is made up of two chromatic components (the *a*^*∗*^component from green to red and the *b*^*∗*^component from blue to yellow, usually ranging from −120 to +120) and a luminance or brightness component (*L∗* value, ranging from 0 for black to 100 for white) [33]. Table 1 presents data found in the selected literature for color value of ﬂours.

*(1) *L*^*∗*^ Value*. *L*^*∗*^ is an important characteristic for drying treatments since it is often the first quality feature that consumers evaluate when determining brand sensory acceptability [34]. Buckman et al. [20] investigated the effects of peeling, blanching, and sodium metabisulfite pretreatments on the ease of drying, particle size distribution, color, and pH of yam bean flour. The results obtained showed that peeling yam samples with sodium metabisulfite pretreatment produced flour samples with the highest *L*^*∗*^ values. This suggests that the sodium metabisulfite pretreatment resulted in some bleaching, which was responsible for the flour's resistance to enzymatic browning. A similar study was reported by Desalegn and Kibr [23], who noticed that anchote flours treated by potassium metabisulfite could increase the whiteness value (*L*^*∗*^) (75.48–84.29), compared to the blanching method and untreated flour. The potassium metabisulfite-treated anchote flour had the greatest whiteness index in this investigation, which could be due to the method's ability to slow both enzymatic and nonenzymatic processes in the anchote flour.

Chemical pretreatments, particularly those involving sulfite and salts, are important to preserve the color of fruits and vegetables due to their potential to retard both enzymatic and nonenzymatic reactions [35]. Furthermore, the same trend of *L*^*∗*^ value increase (which ranged from 54.68 to 50.14) was reported for purple-fleshed sweet potato pretreatment by soaking chips in sodium metabisulfite solution [22]. Sun et al. [36] also indicated that untreated potato samples had a low whiteness color while the citric acid-treated samples had the highest whiteness value due to the color-protecting effect of citric acid. These authors also revealed that a long blanching time increased the *L*^*∗*^ value of the pretreated potato samples. Similarly, Nascimento and Canteri [24] indicated that the *L*^*∗*^ value of unblanched potato flour was darker than the blanched flour. The presence of PPO and peroxidase (POD) enzymes, which are responsible for the enzymatic browning response, results in the creation of dark chemicals contributing to the low *L*^*∗*^ value of the potato sample. However, Rita et al. [6] found that the untreated sample had a higher *L*^*∗*^ value than the boiled, grated, and steamed yam samples.

*(2) *A*^*∗*^ and *B*^*∗*^Values*. The pretreatment methods could decrease the redness/green (*a*^*∗*^) values of the samples. For instance, the untreated anchote flour had a higher value than the blanched and potassium metabisulfite-treated anchote flour dried under different drying methods. The *a*^*∗*^value for anchote flour ranges from 3.02 to 6.34 [23]. Similar to this study, the control sweet potato samples showed a higher redness (*a*^*∗*^ value) than the CaCl_2_- and NaHSO_3_-treated sweet potato samples [37]. However, sulfites can reportedly cause health problems such as asthmatic reactions in sensitive individuals [32]. CaCl_2_ treatment, on the other hand, was the most effective in stabilizing the surface, freshness, and color, had no effect on human health, and was preferred as an alternative chemical treatment method [38].

According to Buckman et al. [20], sodium metabisulfite-pretreated yam flour had the lowest b^*∗*^ value compared to other treatments including blanching and untreated samples. The metabisulfite-treated *Pachyrhizus erosus* flour (jicama) was less yellow than the other samples. The *b*^*∗*^ values (18.26–22.41) for the pretreated sweet potato flour were higher than the control sample at all pretreated concentrations [21]. These highest values were due to the higher amount of beta-carotene in sweet potato flour [37]. According to this study, increasing the concentration of sodium metabisulfite (5–15 g/ml) and citric acid (5–15 g/ml) during the treatment of the sample would increase the yellowness value of the sweet potato flour. Chemical treatments, such as sulfite and salt treatments, are most effective to retard enzymatic and nonenzymatic browning reactions. Likewise, they have antimicrobial properties and protect food components liable to oxidation [39]. The color differences between the flours could also be due to intrinsic pigments, such as beta-carotene, which vary depending on the plant's botanical origin and flour composition [40].

### 2.2. Pretreatment Effect on the Proximate Composition of Tuber Crops

Table 2 presents the findings of different studies regarding the proximate composition of different tuber crops such as moisture, fiber, ash, protein, fat, and carbohydrates.

#### 2.2.1. Moisture Content

The moisture content of all the flours was below the 14% specified standard of the revised regulation of the Standard Organization of Nigeria [41]. The pretreatment process has a significant impact on the moisture content. For example, sweet potato samples pretreated with citric acid at 10 g/L concentration had higher moisture content (7.70%), whereas sodium metabisulfite pretreatment at 15 g/L concentration led to the lowest moisture content (5.54%) [21]. The lower moisture content could be related to the sodium metabisulfite capacity to make sweet potato slices dehydrate by inducing changes in the permeability of the cellular membranes [42]. Desalegn and Kibr [23] also reported that the moisture value for the chemically treated anchote flour samples had lower moisture content than that of hot water blanched and unblanched samples.

The application of pretreatment helps to restrict the moisture losses and improves the drying characteristics of the tuber crops [43]. The authors also found that the moisture content for untreated cocoyam was 6.6 to 11.15%, which was higher than that of the pretreated samples, with the lowest moisture content being 5.88–10.2% for sulfite, 5.97–10.63% for blanched samples, and 5.43–9.67% for combined sulfiting and blanching sample pretreatment methods.

#### 2.2.2. Crude Protein Content

Food proteins are essential nutrients for structural and functional purposes in the human body, and they provide the essential amino acids required for metabolism. Tubers are low in protein content [44]. Pretreatments applied before flour conversion had a considerable impact on protein concentration. In line with this, the sweet potato pretreated by salting, citric acid, and blanching methods had higher crude protein content than the control samples [16]. The same authors also showed that the highest crude protein content was recorded for sweet potato treated with citric acid and then followed by salting and blanching. However, blanching methods cause leaching losses of nutrients, including proteins, from the samples during treatments [45].

Desalegn and Kibr [23] found that anchote samples pretreated with 0.2 N potassium metabisulfite (for 10 minutes) and blanching with hot water (at 70°C for 3 minutes) had the highest crude protein when compared with the control sample. This could be because blanching causes protein solubility in the anchote tuber.

#### 2.2.3. Ash Content

The total ash content of the samples indicates the minerals present [46]. Ngoma et al. [21] found that sweet potato treated with citric acid and sodium metabisulfite at varied concentrations had higher ash content than the other samples. This might be due to the movement of solutes from sodium metabisulfite solution to sweet potato slices during soaking. However, the tuber crops treated with the blanching method had low ash content due to leaching losses during immersion in the hot water [16]. Wibowo et al. [47] found that potato samples treated under steam blanching had higher mineral content than hot water blanching. Chen et al. [48] also confirmed that blanching will result in starch leaching from the cell structures, gelatinization, and breakdown.

#### 2.2.4. Fiber Content

Fibers are nondigestible carbohydrates, which provide fecal bulkiness and less intestinal transit, and play a great role in cholesterol-level reduction [49, 50]. Desalegn and Kibr [23] indicated that the fiber content was measured from 3.22 to 4.71% for the anchote flour treated under different pretreatment methods. The results reported by the aforementioned authors indicated that blanching could increase the fiber content. This could be due to soluble components lost during the application of hot water, thereby increasing the crude fiber contents [51]. Similarly, blanching and boiling of anchote were found to improve the percentage of macromolecules rich in dietary fiber while decreasing the loss of water-soluble components [52]. Another researcher Fana Haile et al. [16] reported that fiber content values of sweet potato were ranged from 3.73 to 7.30%. This study revealed unblanched sample rated highest value, while the chemically treated (salting and citric acid) had the lowest fiber content. Similar to this trend, unblanched plantain sample rated highest fiber content than the sample treated by blanching at 80°C for 10 min followed by sodium metabisulfite-treated samples, while the lowest value was for sample treated with a combination of blanching and sodium metabisulfite treatments. This implies that pretreatment reduces the fiber content [53]. Since obesity, constipation, gallstones, diabetes, and coronary heart disease are all conditions generally reduced when an adequate amount of dietary fiber is consumed [54], these findings show that landraces and processing conditions can generate flour with a higher dietary fiber content, which is beneficial to consumers' health.

#### 2.2.5. Fat Content

Fats are used as an alternative energy source and are essential to the structure and biological processes of cells. Ngoma et al. [21] reported that the untreated sweet potato sample had low-fat content (0.69%) than samples treated by sodium metabisulfite (0.68–2.87%). However, Ahmed et al. [37] indicated that sodium metabisulfite does not influence the lipid content of sweet potato flour. On the other hand, citric acid treatments might lower fat content in Ndou sweet potato flour samples, which ranged from 0.68 to 0.61%. Furthermore, pretreatments could reduce the fat content of anchote flour from 0.72 to 1.14%. For instance, the untreated anchote flour scored the highest fat content than the chemically treated (0.2 N potassium metabisulfite for 10 minutes) and the hot water treated (blanched at 70°C for 3 minutes). The reduction of fat content in the blanched tubers might be due to oxidation [16]. Girma et al. [55] also confirmed that low-fat content will extend the shelf life of food products by reducing the risk of rancid taste development.

#### 2.2.6. Carbohydrate Content

Carbohydrates are among the most important constituents of tuber crops as sources of dietary energy [56]. It was found that the blanching methods of pretreatment could increase the carbohydrate contents of anchote flour than chemical treatments and untreated samples [23]. Bikila et al. [52] also reported that blanching and boiling increase the carbohydrate level of the anchote flour. Shebabaw [57] also found that the carbohydrate content of two anchote accessions increased by boiling from 77.31 to 78.72% for Nekemte, and 77.95 to 79.35% for Dembidolo. Similarly, blanching and drying the sweet potato flour at high temperature enhance the high carbohydrate content [16].

On the other hand, chemical treatment increased the protein, fat, and ash contents of the samples, whereas during blanching these nutrition elements were reduced due to leaching [16, 21, 23]. However, the carbohydrate content of the flour was determined by the percentage differences of moisture, protein, fat, and ash contents, indicating an increase of these parameters due to the decrease of carbohydrates by the chemical treatment.

### 2.3. Pretreatment Effect on the Functional Properties of Tuber Crops

#### 2.3.1. Bulk Density

The bulk density is a measurement of the relative volume of packaging material that is used to determine the storage, transportation, marketing, and wet food processing industries [21, 58]. It also offers scientific information on a product's porosity, which might influence package selection and design [14]. The bulk density characteristic of the flours was affected by the pretreatment processes. The bulk density variation was primarily attributable to the particle size and initial moisture content of the flours [59].

A different bulk density was observed for various tuber crops due to the difference in variety, pretreatment, blanching time and temperature, and types of drying method [16]. The findings reported by Ngoma et al. [21] highlighted the effect of chemical treatment concentration on the sweet potato flour samples. They obtained a low bulk density value of 0.8 g/mL for hot distilled water-treated samples, and the highest values were for samples treated with 5, 10, 15 g/L of citric acid for 10 min and 5, 10, 15 g/L of sodium metabisulfite for 10 min, ranging from 0.86–0.87 g/ml. The results showed that chemical treatments, particularly sodium metabisulfite treatment, could increase the bulk density values of the sweet potato (Table 3). The flour's high bulk density value was critical for food preparations to reduce paste thickness in food products [59].

Desalegn and Kibr [23] also compared the bulk density values of anchote flour treated under different pretreatment and drying methods ranging from 0.71 to 0.76 g/ml. The results reported by the same authors indicated that the treated flour was significantly higher than untreated anchote flour. However, there was no significant (*p* > 0.05) difference between the blanching method and potassium metabisulfite-treated samples (Table 3). According to the same authors' findings, the treated flour had a much higher bulk density than the untreated anchote flour. However, there was no statistically (*p* > 0.05) variation between the blanched and potassium metabisulfite-treated samples. Bikila et al. [52] also confirmed that blanching and boiling of anchote flour could increase the bulk density values than untreated samples.

#### 2.3.2. Water Absorption Capacity

Increased water absorption capacity (WAC) during the preparation of food products could boost yield, uniformity, and food body [61]. As indicated in Table 3, the influence of pretreatment and drying procedures on the quality of anchote flour was explored by Desalegn and Kibr [23]. According to the findings, the treated samples had a better WAC. For example, blanching (2.38–2.56 g/g) and potassium metabisulfite-treated (2.42–2.66 g/g) samples had higher WAC than the untreated anchote flour (2.04–2.66 g/g). The high WAC flours may be used in the production of some bakery products or as a thickener in liquid and semi-liquid foods [58, 62, 63].

Fana Haile and Fisseha [16] determine the WAC of orange flashed sweet potato (OFSP) flour for the samples treated under different pretreatments and drying methods, which were ranged between 2.81–3.9 ml/g. Yusuf et al. [64] highlighted that blanched sun-dried flour had the lowest WAC. This could be because of the high polar amino acid residue of protein with affinity for water. The result indicated by the aforementioned authors is that the salt-treated fluidized bed-dried OFSP flour had higher WAC, whereas the blanched sun-dried wheat had the lowest value.

As reported by Ngoma et al. [21], sweet potato flour pretreatment with various concentrations of sodium metabisulfite and citric acid had a WAC ranging from 1.63 to 2.03 mL/g, while distilled water-treated samples had a WAC of 1.44 mL/g as shown in Table 3. This study indicated that the pretreated flour samples with sodium metabisulfite and citric acid had significantly higher WAC than the control samples that would be useful for bakery products. The enhancement could be attributed to hydrophilic components such as polar or charged side chains of proteins and carbohydrates, which are the key chemical components that boost water absorption ability [65].

#### 2.3.3. Oil Absorption Capacity

Oil absorption capacity (OAC) is an important flour quality characteristic to maintain flavor and improve the mouth feel of the food products [66]. The physical trapping of oil and the binding of fat to a polar chain of the protein are thought to be the key mechanisms of fat absorption [14]. Desalegn and Kibr [23] reported that the OAC value of anchote flours ranged from 1.71 to 2.07 ml/g (Table 3). The blanched and oven-dried samples had the lowest OAC (1.71 ml/g), which was a significantly different value from the highest value of 2.07 ml/g for the potassium metabisulfite-treated and solar-dried anchote samples. The authors also found that blanching treatments could significantly decrease the OAC of the anchote flour. This could be due to oxidation, which results in rancidity. On the other hand, the increased OAC of the flours suggested that polar amino acids were present [58]. The flours with a high OAC are ideal for enhancing flavor and tongue feel [67].

#### 2.3.4. Swelling Power

Swelling power (SP) is a measure of the granules' absorption index after heating and determines the capacity of starch molecules to hold water via hydrogen bonding, which is related to amino acid and starch concentration [68]. Zhu [69] indicated that increasing the temperature could increase the SP of the flour, through disruption and hydration of the crystalline part of the starch leading to granule rupture, consequential in an amorphous state of the starch chains.

According to Rosida et al. [70] findings, all native yam flour has lower SP than modified flour. Specifically, treatments of the yam tuber slice under autoclaving-cooling treatment and heating treatment could increase the SP of yam samples. This could be due to the leaching of the straight-chain amylose during heat treatment [26]. Jangchud et al. [71] also showed that the SP of sweet potato flour was increased as the temperature increased.

Ngoma et al. [21] indicated that the SP of pretreated sweet potato samples (8.47–6.30 g/ml) was significantly higher than that of control samples (7.18 g/ml) (Table 3). This increment of the SP might be due to structural changes within the starch granules. Desalegn and Kibr [23] also indicated that the blanched and chemically treated anchote samples dried under different drying methods had higher SP than untreated anchote flour. The amylose and amylopectin values found in the pretreated tubers corps varied due to the different processing methods. The reduction due to leaching of the straight-chain amylose found in the sample was responsible for the lower SP.

## 3. Conclusion

In this review, impacts of pretreatment techniques on the quality of tuber flour particularly on the physical, functional, and proximate composition were summarized. The reviewed results showed that the application of pretreatments, particularly blanching, can enhance the flour lightness color that is attractive for the consumer. In addition, the pretreatment method can also enhance the dietary fiber, ash, and carbohydrate content of tuber flours. The functional properties of the tuber crop flour were also improved by the pretreatment methods, which is important for the production of valuable food products.

## Figures and Tables

**Table 1:** Review on the effects of pretreatments on the physical properties of tuber.

Tuber crops	Pretreatment methods	Physical properties of the tuber flours						Authors
		*L* ^*∗*^	*a* ^*∗*^	*b* ^*∗*^	Δ*E*	HUE	pH	
*Yam (D. bulbifera)*	Control	85.01	−1.04	11.30	15.01	—	6.65	Buckman et al. [20]
	Peeled and blanched at 100^o^C for 3 min	83.10	−1.46	14.18	18.91	—	6.42	
	Peeled and 0.1% sodium metabisulfite treated for 3 min	90.89	−1.45	10.63	10.90	—	6.13	

*Sweet potato (I. batatas)*	Distilled	80.71	1.27	18.26	—	—	—	Ngoma et al. [21]
	5,10 and 15 ml/g sodium metabisulfite treated for 10 min	83.58–86.79	−0.16–0.80	20.04–21.23	—	—	—	
	5,10 and 15 ml/g citric acid treated for 10 min	69.27–78.82	1.59–3.74	20.88–22.41	—	—	—	

*Sweet potato (I. batatas)*	Control	50.14	10.00	4.16	—	22.62	—	Ulfa et al. [22]
	Steam blanched for 5 min	48.25	9.22	3.19	—	19.13	—	
	2000 ppm sodium metabisulfite treated for 15 min	54.68	10.96	2.64	—	13.55	—	
	200 ppm sodium metabisulfite treated for 15 min and steam blanched for 5 min	52.21	11.16	3.21	—	17.78	—	

*Anchote (C. Abyssinica)*	Untreated and sun-, solar-, and oven-dried	75.48–78.81	4.18–6.34	17.84–18.66	—	70.05–77.18	—	Desalegn and Kibr [23]
	Blanched at 70°C for 3 min and sun-, solar-, and oven-dried	79.71–82.44	3.79–4.41	20.70–21.43	—	73.82–79.88	—	
	0.2 N potassium metabisulfite treated for 10 min and sun-, solar-, and oven-dried	80.83–84.29	3.02–4.36	19.45–20.49	—	77.40–81.61	—	

*Potato (S. tuberosum)*	Unblanched	46.72	2.69	8.20	—	—	—	Nascimento and Canteri [24]
	Blanched at 97°C for 5 min	55.29	1.24	6.09	—	—	—	

**Table 2:** Review on the effects of pretreatments on the proximate composition of tuber crop.

Tuber crops	Pretreatment methods	Proximate composition of the tuber flour						Authors
		Moisture (%)	Protein (%)	Fiber (%)	Ash (%)	Fat (%)	Carbohydrate (%)	
*Potato (S. tuberosum)*	Blanched at 97°C for 5 min	4.48	2.4	4.06	6.91	—	80.67	Nascimento and Canteri [24]
	Unblanched	3.25	1.7	6.28	6.95	—	83.02	

*Sweet potato (I. batatas)*	Control, sun-, solar-, and fluidized bed-dried	5.11–7.36	4.39–5.06	4.12–7.30	5.14–5.68	1.48–2.55	80.62–82.68	Fana et al. [16]
	Blanched at 60°C for 5 min and sun-, solar-, and fluidized bed-dried	5.46–7.79	4.10–4.34	3.84–7.07	4.15–5.10	0.90–1.17	82.79–84.20	
	1% salt treated for 20 min and sun-, solar-, and fluidized bed-dried	4.04–6.78	4.45–5.18	4.17–4.63	6.78–7.51	1.26–1.42	80.01–82.01	
	0.5% citric acid treated for 20 min and sun-, solar-, and fluidized bed-dried	4.38–7.13	5.44–5.76	3.73–4.78	5.25–5.96	1.40–1.89	80.30–82.50	

*Sweet potato (I. batatas)*	Distilled	6.50	2.64	—	1.03	0.69	—	Ngoma et al. [21]
	5, 10, and 15 ml/g sodium metabisulfite treated for 10 min	5.54–6.85	0.68–2.62	—	0.70–2.54	0.68–2.62	—	
	5, 10, and 15 ml/g citric acid treated for 10 min	5.98–7.70	2.54–2.72	—	1.04–1.08	0.61–0.68	—	

*Anchote (C. abyssinica)*	Untreated and sun-, solar-, and oven-dried	9.04–10.34	3.27–3.33	3.22–3.84	4.83–4.79	0.91–1.14	80.95–82.37	Desalegn and Kibr [23]
	Blanched at 70^o^C for 3 min and sun-, solar-, and oven-dried	8.91–9.92	3.14–3.22	3.92–4.71	3.96–3.99	0.72–0.92	81.95–83.03	
	0.2 N potassium metabisulfite treated for 10 min and sun-, solar-, and oven-dried	8.71–9.16	3.76–4.02	3.57–3.90	4.82–4.89	0.77–0.88	81.72–81.92	

**Table 3:** Review on the effects of pretreatments on the functional properties of tuber crop.

Tuber crops	Pretreatment methods	Functional properties of the tuber flour				Authors
		Bulk density (g/m)	Water absorption capacity (g/g)	Oil absorption capacity (ml/g)	Swelling power (g/g)	
*Sweet potato (I. batatas)*	Control, sun-, solar-, and fluidized bed-dried	0.71–0.74	2.90–3.43	0.55–0.75	—	Fana Haile and Fisseha [16]
	Blanched at 60°C for 5 min and sun-, solar-, and fluidized bed-dried	0.71–0.73	2.81–3.38	0.71–1.03	—	
	1% salt treated for 20 min and sun-, solar-, and fluidized bed-dried	0.71–0.73	3.42–3.90	0.66–0.94	—	
	0.5% citric acid treated for 20 min and sun-, solar-, and fluidized bed-dried	0.71–0.73	3.18–3.78	0.6–0.81	—	

*Anchote (C. abyssinica)*	Untreated and sun-, solar-, and oven-dried	0.71–0.73	2.04–2.38	1.71–1.92	9.39–9.61	Desalegn and Kibr [23]
	Blanched at 70°C for 3 min and sun-, solar-, and oven-dried	0.72–0.75	2.38–2.56	1.71–1.92	9.86–12.60	
	0.2 N potassium metabisulfite treated for 10 min and sun-, solar-, and oven-dried	0.73–0.76	2.42–2.66	1.93–2.01	9.46–10.84	

*White yam (D. rotundata)*	Blanched at 70°C for 15 min and sun- and oven-dried	0.86–0.88	2.02–2.28	7.71–7.72	—	Wahab et al. [60]
	0.28% potassium metabisulfite treated for 5 min and sun- and oven-dried	0.83–0.88	1.90–2.40	7.78–7.79	—	

The authors received no financial support for the research authorship.
